# Computational Investigation of Locked Nucleic Acid (LNA) Nucleotides in the Active Sites of DNA Polymerases by Molecular Docking Simulations

**DOI:** 10.1371/journal.pone.0102126

**Published:** 2014-07-18

**Authors:** Vasanthanathan Poongavanam, Praveen K. Madala, Torben Højland, Rakesh N. Veedu

**Affiliations:** 1 Nucleic Acid Center and Department of Physics, Chemistry and Pharmacy, University of Southern Denmark, Odense M, Denmark; 2 Institute of Molecular Bioscience, The University of Queensland, Brisbane, Queensland, Australia; 3 School of Chemistry and Molecular Biosciences, The University of Queensland, Brisbane, Queensland, Australia; Wake Forest University, United States of America

## Abstract

Aptamers constitute a potential class of therapeutic molecules typically selected from a large pool of oligonucleotides against a specific target. With a scope of developing unique shorter aptamers with very high biostability and affinity, locked nucleic acid (LNA) nucleotides have been investigated as a substrate for various polymerases. Various reports showed that some thermophilic B-family DNA polymerases, particularly KOD and Phusion DNA polymerases, accepted LNA-nucleoside 5′-triphosphates as substrates. In this study, we investigated the docking of LNA nucleotides in the active sites of RB69 and KOD DNA polymerases by molecular docking simulations. The study revealed that the incoming LNA-TTP is bound in the active site of the RB69 and KOD DNA polymerases in a manner similar to that seen in the case of dTTP, and with LNA structure, there is no other option than the locked C3′-*endo* conformation which in fact helps better orienting within the active site.

## Introduction

**Figure 1:**
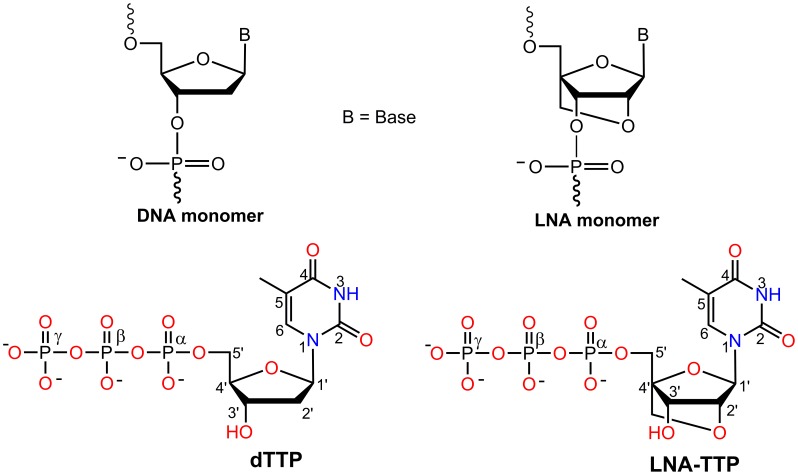
Structural representation of DNA and LNA nucleotide monomers and triphosphates.

Aptamers constitute a class of oligonucleotides selected from a large library pool against a specific target of interest [1]–[5]. The first FDA approved therapeutic aptamer for clinical use is Macugen (Pegaptanib sodium) for the treatment of age related macular degeneration (AMD) [6], [7]. Aptamers containing natural DNA or RNA nucleotides have some serious limitations like poor nuclease resistance (low biostability) and low target binding affinity. Introducing chemically modified nucleotides to aptamers at various positions may help to overcome these problems. Locked nucleic acid (LNA) is one of the most prominent and successful among these analogues and is used extensively for various applications in chemical biology [8]–[11]. LNA nucleotides are generally considered to be RNA mimicking molecules in which the ribose sugar moiety is locked by an oxymethylene bridge connecting the C2′ and C4′ carbon atoms, imposing conformational restriction to adopt C3′-endo/*N*-type furanose conformation (Figure 1) [12]–[14]. LNA offers unique properties needed for successful therapeutic application of oligonucleotides such as high binding affinity to complementary DNA and RNA oligonucleotides and high stability in biological systems, *ie*. resistance towards enzymatic degradation. The usefulness of LNA-modified oligonucleotides for various applications has been the subject for many scientific investigations [10], [11]. With a scope of developing unique shorter aptamers with very high biostability and affinity, locked nucleic acid (LNA) nucleotides have been investigated as a substrate for various polymerases.

Recently, we and others have reported on the ability of polymerases to accept LNA nucleoside 5′-triphosphates, LNA-TTP (Figure 1) as substrates [15]–[23]. B-family DNA polymerases like KOD, Phusion and 9^o^N_m_ were shown to be able to recognize LNA-triphosphates compared with A-family polymerases. Based on the amino acid sequence similarities to *E. coli* polymerases I, II, III, and IV/V DNA polymerases are classified into four families such as A, B, C and Y [24], [25]. Family A and B DNA polymerases share comparable structural elements like a recognizable finger, thumb, and palm subdomains although the amino acid sequences differ [26], [27]. In addition, the interactions of two divalent metal ions in the polymerase active sites are also important to catalyse a phosphoryl transfer reaction in order to incorporate dNMPs into DNA [26], [27]. KOD DNA polymerase was found to be the most suitable enzyme to read and incorporate LNA nucleotides. Højland *et al.* recently reported that a DNA mimicking alpha-L-LNA nucleotide, a diastereomer of LNA, can also serve as a substrate of polymerases like Phusion, KOD and 9^o^N_m_ [28]. It is highly surprising that the conformationally constrained LNA nucleotides are compatible with DNA polymerase activity. KOD and RB69 DNA polymerases belong to Family B type and also have a very high sequence and structural homologies providing an excellent framework for this investigation [29]. Conformational flexibility is an important factor for a nucleotide to serve as substrates for polymerases. Despite this, LNA nucleotide as one of the prominent example of conformationally constrained nucleic acid analogues is well tolerated by the B-family polyemarses like KOD, Phusion and 9^o^N_m_. To evaluate and gain further insights of these remarkable experimental findings, it is important to perform a structural investigation. For this purpose, we initiated a molecular docking simulation approach using recent crystal structure of a polymerase complexed with an incoming DNA nucleoside triphosphate and primer-template DNA duplex. Herein, we report molecular docking studies of the LNA-triphosphate (LNA-TTP) in the active sites of RB69 and KOD DNA polymerases including the effect of terminal LNA-nucleotide modified primer and template.

## Materials and Methods

### Preparation of Protein and Ligands

#### Preparation of LNA nucleoside 5′-triphosphate ligand

The ligand was built using the ChemBioDraw version 13.0 [30] and imported into Maestro module in Schrödinger suite [31]. Subsequently, the ligand was pre-processed using the LigPrep module of the Schrödinger package, pre-processing includes 3D conformation generation (using the OPLS2005 force field) and ionization states for the ligand at pH 7.0 −/+2.0 were predicted using the Epik tool [32]. Epik is an application that generates possible protonation states, tautomers and metal binding sites in the ligand. Stereochemistry for the ligand kept unchanged during the ligand preprocessing. The ligand was energy minimized using semi-empirical method (AM1 method) with RHF wave function as implemented in MacroModel module of Schrödinger suite.

#### Preparation of Protein and Grid Generation

The structural coordinates for RB69 DNA polymerase complexed with duplex DNA and the incoming dTTP (2′-deoxythymidine triphosphate, dTTP; Figure 1) were obtained from the crystal structure PDB ID: 1IG9 [33] (with resolution of 2.60 Å). Also, the terminal nucleotide on the primer DNA strand was structurally modified to an LNA nucleotide using the Maestro module in Schrödinger suite. The final model with LNA-modified DNA strand was further refined to remove steric clashes within the molecule by minimisation as described above. The protein was further optimized using the Protein Preparation Wizard [34]. This optimization includes adding hydrogen atoms, assigning correct bond orders and building di-sulfide bonds. The protonation states of all of the ionizable residues were predicted by PROPKA [35] provided in the Protein Preparation Wizard in the presence of the Ca^2+^ ions at the active site. An optimized structure model was energy minimized (only hydrogen atoms with converge heavy atoms to RMSD below 0.3 Å) using the OPLS2005 force field. Active site water molecules within 3 Å from the bound ligand were kept for the docking simulation and remaining were removed. The orientation of hydrogen atoms in the water was sampled using PROPKA [35].

The structural coordinates for KOD DNA polymerase complexed with duplex DNA were obtained from the crystal structure PDB ID: 4K8Z [36] (with resolution of 2.29 Å). KOD-dTTP model was built based on the 1IG9 coordinates using Prime module (a homology modelling tool) of Schrödinger suite, as there was no structure available with the incoming triphosphates for KOD DNA polymerase. The KOD-dTTP model was pre-processed as described for RB69. Same protocol was used for each modified primer-template with a terminal LNA nucleotide and the incoming triphosphate complex.

The receptor grid generation module of Glide [37] was used to define the active site for the docking experiments. As this protein model has a bound ligand (dTTP), the ligand was set as the centroid of the grid box (size of the active site is 20 Å from ligand position). Same protocol was used for modifying the primer and template with a terminal LNA nucleotide in the active site of RB69 DNA polymerase and KOD DNA polymerase.

#### Ligand docking simulation

Glide (version 5.8), a grid-based exhaustive search algorithm was used for all docking experiments [38]. Glide uses a series of hierarchical filters to find possible ligand pose in the active site, and the program has the option to treat the ligand fully flexible (current setting) or rigid during the docking run. Glide uses an in-built docking scoring function resulting in a Glidescore (Standard precision (SP) and extra precision (XP). In the current setting, SP docking modes were used and top 10 binding poses were analyzed after post-minimization process (threshold for rejecting the minimized pose was set to 0.5 kcal/mol).

## Results and Discussion

### Validation of docking simulations

**Figure 2:**
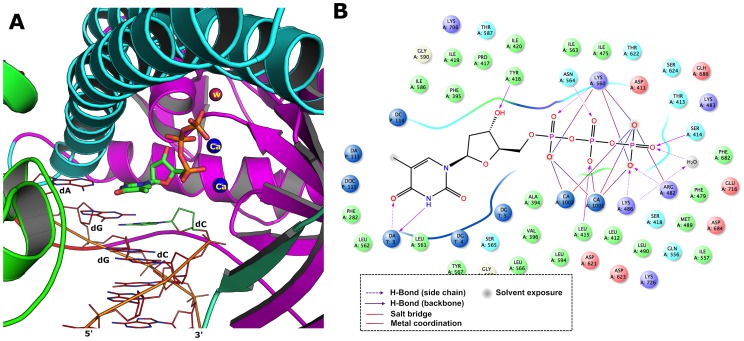
Structure and triphosphate binding interaction of RB69 DNA polymerase. Panel A. Active site shown with bound ligand, catalytically active metal ions and water; Panel B. 2D representation of protein-ligand interaction of bound ligand from docking simulation.

Reproducing the crystallographically observed conformation of the ligand (dTTP) is a minimum requirement to determine whether a docking setup is applicable to a given system. The refined receptor models (for both RB69 and KOD DNA polymerases) were used for all docking simulations. Initially the dTTP was prepared as described in the ligand preparation section and docked using the standard precision mode (SP) into the active site. Subsequently we compared the conformation and position with the bound ligand conformation measured in terms of the root-mean-square-deviation (RMSD). The best 10 poses were analyzed. From the results, all the 10 docking poses were reproduced the crystal bound conformation with a RMSD below 2.0 Å and moreover, the first two ranked poses had a RMSD of 0.5–0.7 Å (Supplementary information, Figure S1). By analyzing the binding mode of first ranked docking pose (Figure 2), the dTTP shows very similar interaction with residues as observed in the reported crystal structures of RB69 DNA polymerase, for instances, nitrogen and oxygen atoms of thymidine ring of dTTP forms hydrogen bonding with terminal adenine base of the template. Hydroxyl group of C3′ carbon in ribose ring make hydrogen bonding with neighboring Tyr416 with a distance of 2.2 Å, which in fact is smaller than the observed distance in the crystal structure (3.17 Å). As shown in the bound conformation, oxygen atoms of the α, β, γ phosphate groups in dTTP also shows metal coordination with two catalytically active Ca^2+^ ions. In addition, a large number of salt-bridges were also found, for example, Lysine 486 and 560 with oxygen atoms of α and γ phosphate group and Arg482 with non-bridging oxygen atoms of the γ phosphate group. Hydrogen bonding interaction from the side chain residues were also significantly found with dTTP particularly, Ser414, Leu415 and Asn564. Moreover, a water molecule at the active site also plays a significant role in the ligand binding as this directly coordinated with ligand a distance of 2.6 Å and Lys486 (makes salt bridge with ligand) with distance of 2.8 Å.

### Binding mode of LNA-TTP in RB69 and KOD DNA polymerase active sites

**Figure 3:**
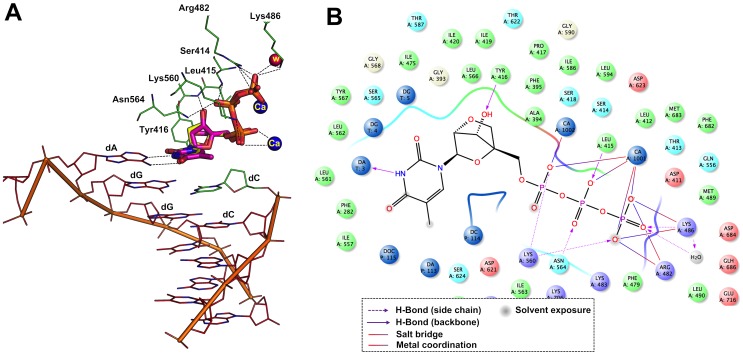
Binding mode of LNA-TTP and dTTP in the binding site of RB69 polymerase. Panel A. Comparison of binding mode of LNA-TTP (pink stick) and dTTP (yellow stick) at polymerase active site (important residues are highlighted including metal and water); Panel B. 2D representation of protein-ligand interaction of LNA-TTP from docking simulation is shown in right panel.

First we performed the docking experiments on RB69 polymerase-DNA complex with incoming nucleoside 5′-triphosphates, dTTP and LNA-TTP ligands (Figure 3). From the analysis it was revealed that the introduction of LNA-TTP did not make any significant changes compared with the natural dTTP in the docked orientation of the incoming nucleotides, other than reorienting the hydrogen bond interactions with other residues within the polymerase active sites. The overall RMSD of LNA-TTP and bound dTTP found to be 0.28 Å (calculated only for similar atom pairs). The triphosphate tail of LNA-TTP interacts with three positively charged residues in the two most conserved motifs of the fingers domain: Arg482 interact with the γ phosphate while Lys560 interact with the oxygen between the β and γ phosphates and Lys486 interact with the oxygen atom bound to the terminal γ phosphate. The ribose of the LNA-TTP stacks on top of the phenyl ring of Tyr416 (in motif A) in a similar fashion as seen with dNTPs [33] and Tyr115 in the ternary complex structure as shown by Huang *et al.* and in the ternary complex structure of HIV-1 RT [38]. The two Ca^2+^ ions coordinate with highly conserved aspartate residues, Asp411 and Asp623 together with the β and γ phosphates of the LNA-TTP. Incoming dNTP and catalytic metal ions binding is key for further rearrangements of the catalytic amino acid residues and proper geometric arrangement of all reacting residues and atoms are thus essential for the formation of the productive ternary complex [39]–[42]. It is very important to mention that the ribose of the LNA-TTP adopts a C3′-endo conformation as observed with dTTP in the RB69 crystal structure [33] (Supplementary information, Figure S2). In addition, hydroxyl group of C3′ carbon in ribose ring of LNA makes hydrogen bonding with Tyr416 with the distance of 2.6 Å as observed in dTTP.

**Figure 4:**
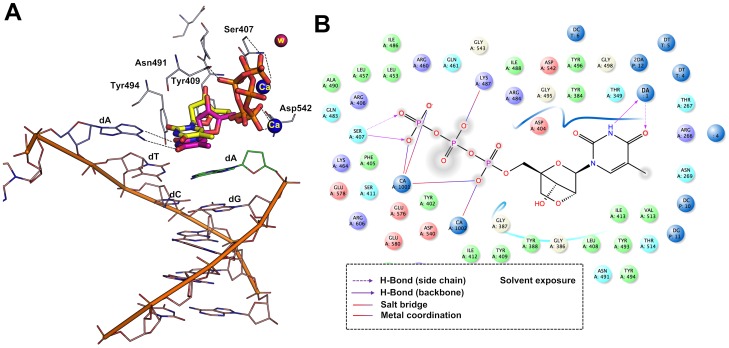
LNA-TTP and dTTP binding mode in the active site of KOD DNA polymerase. Panel A. Comparison of binding mode of LNA-TTP (pink stick) and dTTP (yellow stick) in the KOD polymerase. Active site residues are highlighted including catalytic metal and water; Panel B. 2D representation of protein-ligand interaction of LNA-TTP from docking simulation is shown in the right panel.

It was reported that KOD DNA polymerase, another thermophilic B-family DNA polymerase like RB69, was the best enzyme experimentally observed to accept LNA-TTP as a substrate [17], [19]. To evaluate this, we then performed docking studies on a computational model of KOD DNA polymerase with the incoming nucleoside triphosphates using a recently published crystal structure [36]. The results showed that the triphosphate tail of LNA-TTP hydrogen bonding with Asn491, Asp542 and Arg406 residues and with the non-bridging oxygen atoms of the α, β and γ phosphates respectively (Figure 4). It should be noticed that the thymidine and ribose groups are slightly down positioned compared to the dTTP, however, both the groups are still located within the interaction region with neighboring residues. Hydrogen bonding network between thymidine of LNA with deoxyadenine found to be within 2.5 Å. Furthermore, the hydrogen bonding between hydroxyl group of C3′ carbon in the ribose of LNA and Tyr416 is with the a distance of 3.0 Å. Overall RMSD between dTTP and LNA-TTP found to be 0.9 Å (calculated from similar heavy atom pairs). In addition, the terminal oxygen atoms of phosphates interact with Lys486 and the ribose of the LNA-TTP stack with Tyr416 and Tyr115. These interactions were also in line to previous reports of crystal structure analysis for polymerase docking of nucleotides [33], [38], [43], [44]. Binding mode of dTTP and LNA-TTP in KOD is shown in Figure 4.

### Binding mode of LNA-TTP in primer-template with terminal LNA nucleotides

**Figure 5:**
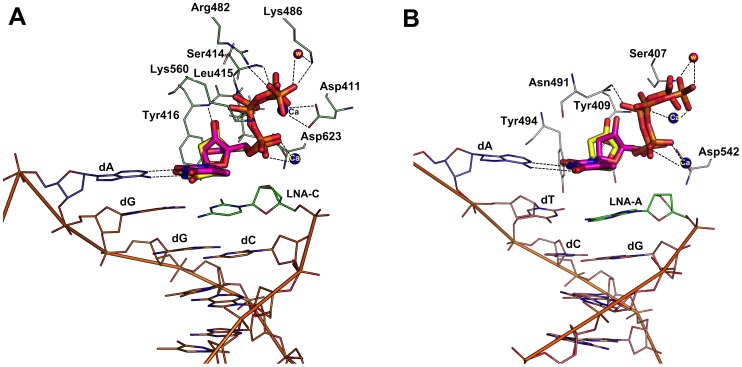
Binding mode of LNA-TTP and dTTP in a primer modified with a terminal LNA-nucleotide in RB69 and KOD DNA polymerase. Panel A. Comparison of binding mode of LNA-TTP (pink stick) and dTTP (yellow stick) in RB69 polymerase; Panel B. Comparison of binding mode of LNA-TTP (pink stick) and dTTP (yellow stick) in the active site of KOD DNA polymerase.

From the literature, it was very surprising to note that thermophilic B-family polymerases like KOD, Phusion and 9^o^N_m_ DNA polymerases can incorporate LNA nucleotides consecutively opposite to natural nucleotides of the template strand and also to incorporate LNA nucleotide opposite to an LNA nucleotide of the template strand [17], [21]. To investigate more about these findings, first we have developed the models using the RB69 and KOD DNA polymerase-DNA crystal structure in which the terminal nucleotide on the primer DNA strand was structurally modified to an LNA nucleotide (described in the method section) in order to understand how this change effects the incoming LNA-TTP and dTTP orientation within the active site. Docking simulation analysis showed that the terminal LNA nucleotide at the 3′-end of the primer DNA did not affect the docking of the incoming LNA-TTP, which was also found to be the same in the case of an incoming dTTP (Figure 5). Later, we performed the same analysis using the crystal structure of KOD DNA polymerase. Again, we found that the introduction of an LNA nucleotide at the 3′-end of the DNA primer did not make any dramatic changes to the active site docking of the incoming LNA-TTP or dTTP in KOD DNA polymerase (Figure 5). We also analyzed the top five docking pose of LNA in this experiment and the result revealed that all the docking poses are also in agreement with the same observation mentioned above (Supplementary information, Figure S3).

**Figure 6:**
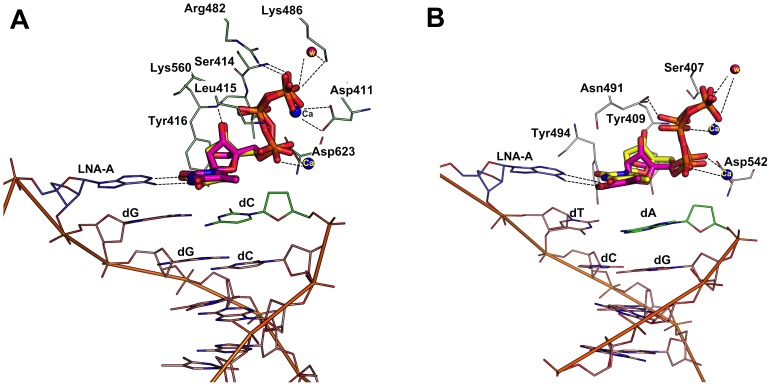
Binding mode of LNA-TTP and dTTP in a template modified with a terminal LNA-nucleotide in RB69 and KOD DNA polymerase. Panel A. Comparison of binding mode of LNA-TTP (pink stick) and dTTP (yellow stick) in RB69 polymerase; Panel B. Comparison of binding mode of LNA-TTP (pink stick) and dTTP (yellow stick) in the active site of KOD DNA polymerase.

**Figure 7:**
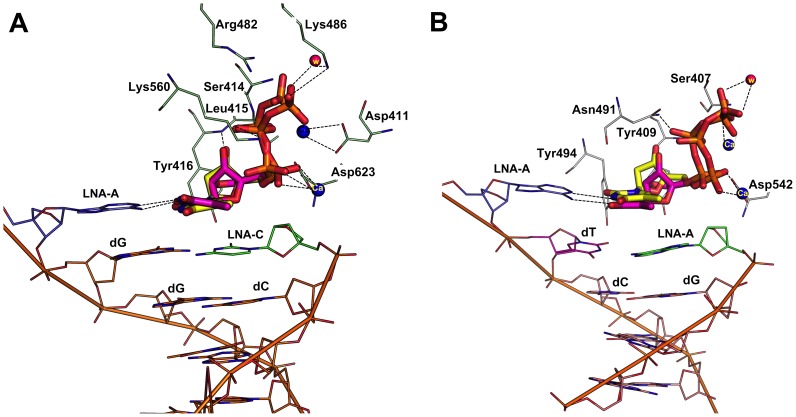
Binding mode of LNA-TTP and dTTP in primer and template modified with a terminal LNA-nucleotide in RB69 and KOD DNA polymerase. Panel A. Comparison of binding mode of LNA-TTP (pink stick) and dTTP (yellow stick) in RB69 polymerase; Panel B. Comparison of binding mode of LNA-TTP (pink stick) and dTTP (yellow stick) in the active site of KOD DNA polymerase.

In addition, this experiment was further extended to analyze the effect of incoming LNA-TTP and dTTP in which the terminal nucleotide on the template DNA strand was modified with an LNA nucleotide. From the analysis, it was observed that the dTTP and LNA-TTP was still able to bind in the active sites of both RB69 and KOD (Figures 6) and involved in similar interactions as observed previously. Furthermore, we also docked dTTP and LNA-TTP using a primer-template complex in which the terminal nucleotide is modified with an LNA nucleotide in both the strands in RB69 and KOD DNA polymerases. Results showed that there is a minor relocation of phosphate tail that significantly changed the type of interactions with active site residues, however, both dTTP and LNA are still able to bind in the modified DNA strand as we observed for the natural DNA primer and template strands (Figure 7).

## Conclusions

In summary, we have investigated the positioning of an LNA-nucleoside 5′-triphosphate in the active sites of RB69 and KOD DNA polymerases by molecular docking simulations. The results clearly show that LNA-TTP docked well in the active site by maintaining the required contacts with the neighbouring amino acids and the catalytically active Ca^2+^ ions for catalysing the nucleotide polymerisations. We speculate that the C3′-endo conformation of LNA-TTP in the active site and positioning may account for better tolerance of LNA nucleotides by B-family polymerases. This study in our view will certainly help to gain more insights on polymerase recognition properties and requirements for chemically-modified nucleotide analogues towards developing chemically modified aptamers by SELEX processes.

## Supporting Information

Figure S1 Panel A. Overall Structural fold and Interaction of dTTP with RB69 DNA polymerase; Panel B. Comparison of dTTP conformation from docking simulation (cyan) with crystal bound conformation (yellow).
(TIFF)

Figure S2 **Binding mode of substrates at RB69 binding site.** Panel A. comparison of binding mode of LNA (pink stick) and dTTP (yellow stick) at polymerase active site (important residues are highlighted including metal and water); Panel B. Interaction of LNA and dTTP with important residues is shown from side view, for better clarity of the furanose ring.
(TIFF)

Figure S3 **Binding modes of top 5 docking poses of LNA-TTP in the RB69 active site.**
(TIFF)
