# Supplementary figures and images for: Computational Investigation of Locked Nucleic Acid (LNA) Nucleotides in the Active Sites of DNA Polymerases by Molecular Docking Simulations
Source: PLoS One. 2014 Jul 18;9(7):e102126. doi: 10.1371/journal.pone.0102126 (PMC4103837; doi:10.1371/journal.pone.0102126)

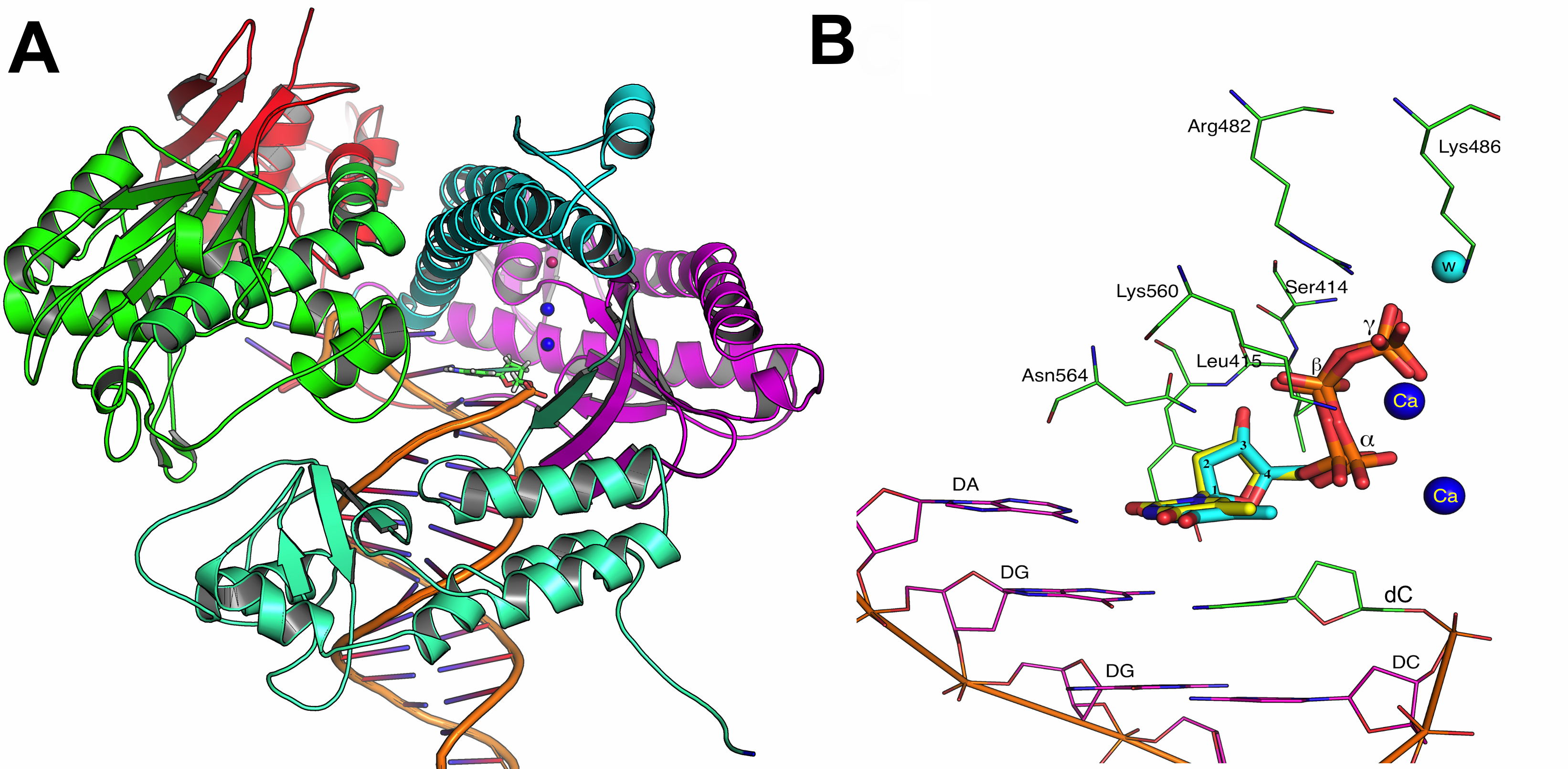

Supplement: Figure S1 — Panel A. Overall Structural fold and Interaction of dTTP with RB69 DNA polymerase; Panel B. Comparison of dTTP conformation from docking simulation (cyan) with crystal bound conformation (yellow). (TIFF) [file pone.0102126.s001.tiff]

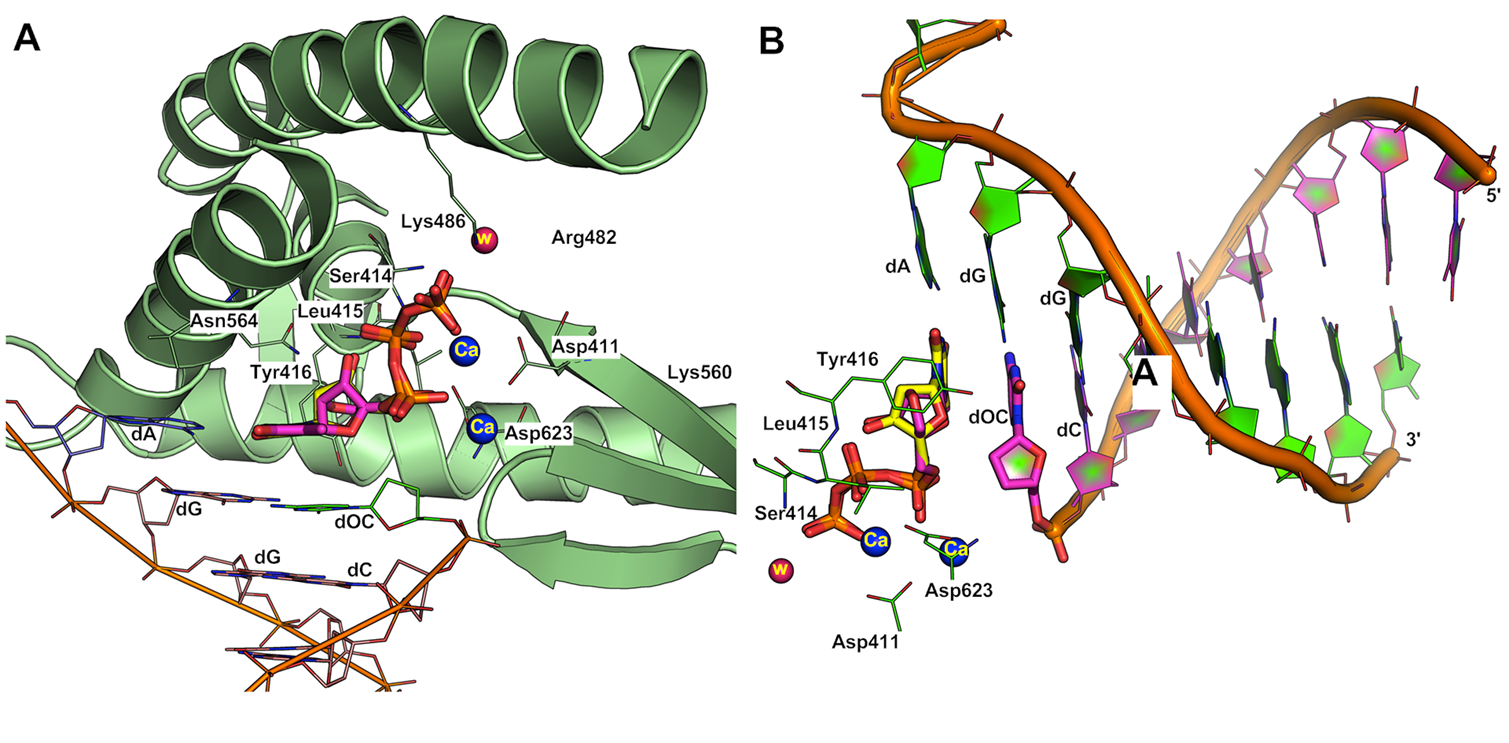

Supplement: Figure S2 — Binding mode of substrates at RB69 binding site. Panel A. comparison of binding mode of LNA (pink stick) and dTTP (yellow stick) at polymerase active site (important residues are highlighted including metal and water); Panel B. Interaction of LNA and dTTP with important residues is shown from side view, for better clarity of the furanose ring. (TIFF) [file pone.0102126.s002.tiff]

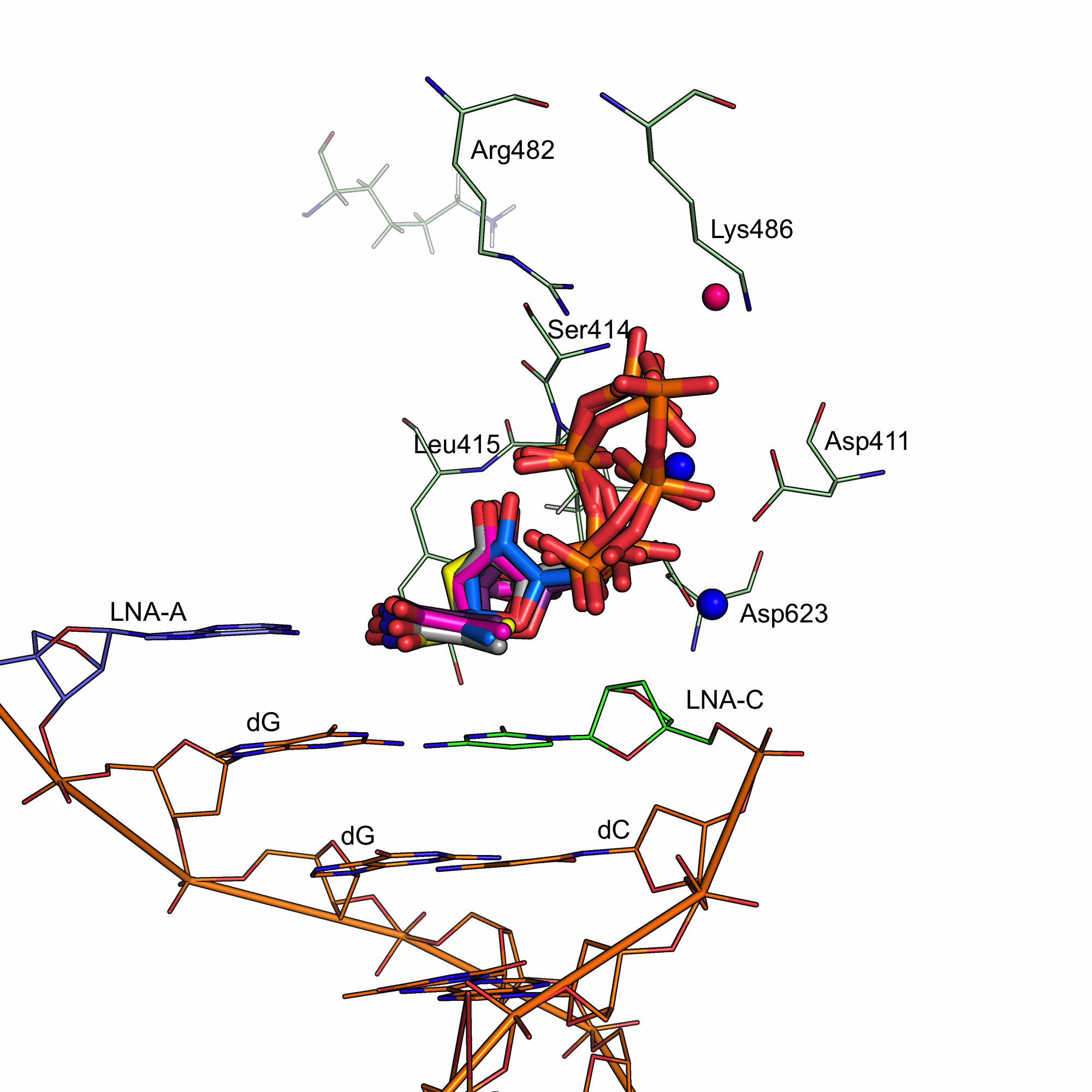

Supplement: Figure S3 — Binding modes of top 5 docking poses of LNA-TTP in the RB69 active site. (TIFF) [file pone.0102126.s003.tiff]
